# The Presidency

**Published:** 1951-01

**Authors:** 


### Editorial

**The Presidency**

Those members of the Bristol Medico-Chirurgical Society who were present at the annual general meeting must have been astonished to hear: " It is a tradition of the Society to choose as President in successive years a physician, a surgeon, a specialist and a general practitioner ". This looks good for the physicians. Anyone who consults the last published list of members* will discover that the chances, for a member of each group, of election to this high and responsible office are about 25 : 13 : 3 : 1. But turn to the list of former Presidents* and it is clear at once that there is no such tradition. We have had two physicians in succession, or two surgeons—even two anaesthetists; but such a sequence as is suggested only once in the last fifty years.

Two traditions, indeed, come down to us from the earliest years of the Society; one, that it accepts the nomination of the Committee, trusting that body to propose the worthiest candidate unhampered by private pacts or secret conventions; the second, that those members who have for several years borne the (often considerable) burden of junior office, are selected for early elevation.† Unless we hold, with Montaigne, that " Dignities and offices are of necessity conferred more by fortune than upon account of merit", the highest honour the Society can bestow should, like a seat in the Prytaneum, reward personal worth and services to the Society—or at the least, long membership and the esteem of colleagues. To say, in effect, " You are not the member we *want*, but this year we are bound to choose a physician", must at one stroke degrade both the man and the office.

* 1948, LXV, 35, 34.

† e.g. Secretary 1874–88, President-elect at age 44; 1894–1902, at 45; 1912–19 at 50; Assistant Editor 1883–89, President-elect at 41; 1900–12 at 51; 1912–22, at 47.

